# Manipulating Air-Gap Electrospinning to Create Aligned Polymer Nanofiber-Wrapped Glass Microfibers for Cortical Bone Tissue Engineering

**DOI:** 10.3390/bioengineering7040165

**Published:** 2020-12-20

**Authors:** Houston R. Linder, Austin A. Glass, Delbert E. Day, Scott A. Sell

**Affiliations:** 1 Biomedical Engineering, Parks College of Engineering, Aviation, and Technology, Saint Louis University, Saint Louis, MI 63103, USA; Houston.Linder@slu.edu (H.R.L.); Austin.Glass@slu.edu (A.A.G.); 2 Material Science and Engineering, Missouri University of Science and Technology, Rolla, MI 65409, USA; Day@mst.edu

**Keywords:** electrospinning, bioglass, 1393 bioactive glass, polycaprolactone, critical size bone defect, cortical bone, osteon, scaffold

## Abstract

Osteons are the repeating unit throughout cortical bone, consisting of canals filled with blood and nerve vessels surrounded by concentric lamella of hydroxyapatite-containing collagen fibers, providing mechanical strength. Creating a biodegradable scaffold that mimics the osteon structure is crucial for optimizing cellular infiltration and ultimately the replacement of the scaffold with native cortical bone. In this study, a modified air-gap electrospinning setup was exploited to continuously wrap highly aligned polycaprolactone polymer nanofibers around individual 1393 bioactive glass microfibers, resulting in a synthetic structure similar to osteons. By varying the parameters of the device, scaffolds with polymer fibers wrapped at angles between 5–20° to the glass fiber were chosen. The scaffold indicated increased cell migration by demonstrating unidirectional cell orientation along the fibers, similar to recent work regarding aligned nerve and muscle regeneration. The wrapping decreased the porosity from 90% to 80%, which was sufficient for glass conversion through ion exchange validated by inductively coupled plasma. Scaffold degradation was not cytotoxic. Encapsulating the glass with polymer nanofibers caused viscoelastic deformation during three-point bending, preventing typical brittle glass fracture, while maintaining cell migration. This scaffold design structurally mimics the osteon, with the intent to replace its material compositions for better regeneration.

## 1. Introduction

Around 1.6 million people require bone grafts annually in the U.S. for degenerative diseases, injuries, tumors, and infections, accounting for approximately USD 244 billion [1,2]. Bone grafts are currently used when this bone injury is larger than what the natural bone healing process can mend, which is termed a critical size bone defect. This is clinically determined when the defect site is twice as large as the diameter of the injured bone [3]. The current gold standard for critical size bone defects involve the use of autologous bone grafts, but donor and receiving sites potentially experience pain, complications, limited donor bone volume, and increased risks of infection [4,5,6,7]. These current methods are lacking, whether it is due to the addition of an invasive surgery inducing another fracture, or risks immune rejection from another donor. Because of this, there has been a rise in interest over the last decade to create bone substitutes that mimic and replace the native bone.

Bones are composed of the inner, spongy trabecular bone, which is surrounded by the outer, dense cortical bone. Cortical bone provides 80% of the bone’s mechanical strength and is made up of repeating functional units, called the osteon. Osteons are hollow cylindrical structures of five to 20 concentric layers of bone tissue, surrounding the haversian canals, which house the blood and nerve vessels. While the haversian canals are approximately 50 μm in diameter, the varying number of lamellae cause different sizes of osteons, but are typically around 200 μm in diameter [8]. Osteogenic cells reside throughout the osteons, causing constant remodeling of the bone tissue, which is composed of collagen and hydroxyapatite (HA). Creating a biodegradable scaffold that mimics the osteon structure should further encourage osteon regeneration by guiding cell migration and promoting angiogenesis [9]. The design presented here utilizes the osteon structure to wrap aligned electrospun polymer nanofibers around bioactive glass microfibers, with the intent to promote directional cell migration and blood vessel infiltration.

Electrospinning is a method of producing highly porous, nano-sized fibers by applying a high voltage to an extruding polymeric solution [10]. The high voltage causes instability and thus rapid spiraling motions from electrostatic repulsion to evaporate the solvent before depositing on the substrate. Typically, this method results in randomly aligned fibers that mimic the extracellular matrix, allowing for better cellular integration into the scaffold [11]. Altering the electrospinning parameters (polymer concentration, voltage, working distance, flow rate, solution conductivity, solvent, humidity, and temperature) changes the properties of the scaffold, such as the fiber diameter, porosity, and mechanical strength, creating a tailorable scaffold [10,12]. Aligning the fibers has been shown to increase directional cell migration, which has been utilized for different cell types such as nerve [13,14,15], blood vessel [16], muscle [17], bone [18], and cartilage regeneration [19]. 

The two most common methods for producing aligned nanofibers are through rapid rotation of the collecting mandrel or through manipulating the electric field [11]. By applying a negative voltage to the polymer solution and a positive voltage to two plates separated by a gap, the fibers are electro-statically attracted to both plates and so they stretch from one plate to the other. This method is termed "air gap electrospinning," and is primarily affected by the polymer concentration, plate distance, plate size, or voltage applied [15,20]. Air-gap electrospinning has a high potential for use in cortical bone, since the aligned electrospun fibers resemble the aligned collagen fibrous layers of the osteon. Furthermore, electrospun scaffolds have been shown to proliferate osteoblasts and mesenchymal stem cells [21,22], with aligned fibers demonstrating organized collagen deposition onto the fibers [23]. Despite these benefits, aligned fibers are limited in perpendicular tension and thus have yet to be used to their full potential. 

The increased cell migration from the aligned electrospun fibers will be limited by nutrient diffusion from the blood vessels to the osteogenic cells. Bioactive glass has been shown to promote blood vessel formation, as well as increasing bone formation quicker than HA alone [24]. This is due to bioactive glass converting to HA while releasing beneficial ions, with the conversion being a dynamic process [25]. In short, hydrolysis occurs on the outside of the glass onto the network modifiers (i.e., Na^+^ and Ca^2+^), ultimately increasing the pH in the surrounding liquid. For silica-based bioactive glasses, this causes the dissolution of silica, which then polymerizes onto the outside of the glass as porous silica-rich layers. As the glass continues to degrade from hydrolysis, the released Ca^+^ and (PO_4_)^3−^ ions travel through the silica rich layer, and will deposit on the outside to form amorphous calcium phosphate. After incorporating (OH)^−^ and (CO_3_)^2−^ from the surrounding solution, it will form a HA-like layer, the primary mineral in bone. The result is a hollow HA structure that maintained the original glass structure. This rate of conversion can be quickened by replacing the silicate with borate [26], or by replacing network modifiers with an alkali ion of larger radius, such as replacing K_2_O with Na_2_O [27].

Certain bioactive glasses have been shown to be osteoconductive as well as osteoinductive, as opposed to HA, which may not promote osteoinduction [25]. This is due to the released calcium and silica during the glass conversion, which induces growth factor adsorption followed by the attachment, proliferation, and differentiation of osteoprogenitor cells [28]. These osteoblasts then secrete collagen onto the surface of the HA-converting bioglass, which the collagen begins to mineralize while being imbedded into the HA layers [29]. Additionally, bioactive glasses have been proven to have angiogenic potential, as expected with the cellular responses of the incorporated elements [24,30,31,32,33,34]. The released ions during conversion to HA simultaneously induce angiogenic cellular responses and pro-osteogenic responses.

Bioactive glass is capable of having strong physical bonds with bone [35], resorbed in 6 months with little inflammatory rate [36], and promotes neovascularization within two weeks [37,38]. However, the use of bioactive glass is limited due to its brittle nature, and so incorporation into a composite is necessary to compensate. The novel design in this paper utilizes a modified version of air gap electrospinning to wrap highly aligned polymer nanofibers around a bioactive glass microfiber. These homologous and uniform synthetic osteons (HOUSteons) can be placed together and electrospun around them in a similar manner, creating a HOUSteon bundle. The design presented here mimics the osteon structure using aligned electrospun nanofibers wrapped around bioactive glass microfibers, to promote directional cell migration with the potential for increasing blood vessel infiltration.

## 2. Materials and Methods 

### 2.1. Design and Optimization of Modified Air-Gap Electrospinning Apparatus

**Figure 1:**
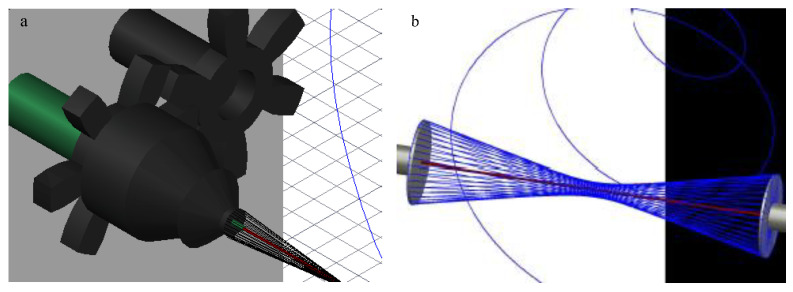
AutoCAD images of (**a**) the final design of the modified air-gap electrospinning with motor setup, signifying the stationary (green) and rotating (black) parts. (**b**) A simplified setup demonstrating the wrapping of electrospun fibers (blue) around the glass fiber (red).

The collecting air-gap design was primarily composed of 3D-printed polylactic acid (PLA) and aluminum foil, which was adhered to the air-gap mandrel for conduction. The final setup is shown using 3D design software (AutoCAD, Autodesk Inc., San Rafael, CA, USA), in Figure 1. The 9V battery-powered 168-RPM motors were connected to gears in order to have a gear rotating around a stationary cylinder. The rotating gear was attached to a metal bearing to simultaneously reduce friction and conduct electricity. This was attached to the conductive air-gap mandrel. The conductive wire was attached to the stationary part of the bearing, allowing electricity to conduct through the metal bearing to the rotating mandrel. The mandrel had a hole in the center for the stationary glass holder so the glass fiber could be placed and held stationary with tacky glue. Both sides are designed the same except the mandrels rotate in opposite directions to induce fiber wrapping.

Initial mandrel testing involved two metal washers with diameters of 31 and 22 mm, followed by a 4 mm PLA cone-shaped mandrel coated in aluminum foil. The 4 mm cone was chosen over its flat counterpart due to more consistent scaffold fabrication. The mandrel distance had no effect on fiber deposition, so the 8 cm maximum distance between the mandrels of this setup was selected. A voltage regulator was used to adjust the rotational speed and direction of the mandrels. The stationary glass holder was tested within the mandrel and extended from the mandrel. 

### 2.2. Fabrication of 1393 Glass Fibers

The bioactive 1393 silicate glass (53 SiO_2_-20 CaO-6 Na_2_O-4 P_2_O_5_-12 K_2_O-5 MgO wt%) was prepared through standard procedures reported elsewhere [26]. In short, the powdered CaCO_3_, Na_2_CO_3_, MgCO_3_, K_2_CO_3_, SiO_2_, and CaHPO_4_·2H_2_O (Fisher Scientific, St. Louis, MO, USA) were mixed thoroughly for 30 min. This batch was melted in a platinum crucible and an electric furnace at 1350 °C for 2 h until homogeneously melted. Once melted, a silica rod was dipped into the solution and raised to produce initial fiber formation. These fibers were pulled by hand, annealing instantly at room temperature. Fibers that were less than 150 μm without bead formation were used in this study.

### 2.3. Fabrication of Scaffold 

The randomly oriented electrospun scaffold was fabricated using a traditional stainless steel rectangular mandrel (9 × 5 × 2.5 cm^3^), rotating at 500 rpm. Polycaprolactone (PCL) with an average molecular weight of 80,000 g/mol (Sigma Aldrich, Milwaukee, WI, USA) at 12 wt% was dissolved in 1,1,1,3,3,3-hexafluoro-2-propanol (HFP) overnight on a shaker plate, and then 3 mL of this solution was electrospun with a flow rate of 3 mL/hr. The voltage was −18 kV on the needle with the mandrel grounded, and the working distance was 15 cm. The scaffolds were stored in a desiccator.

**Figure 2:**
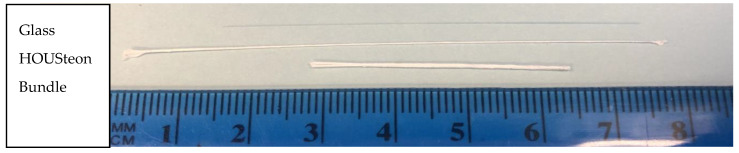
Macroscopic image of a glass fiber, HOUSTeon, and HOUSteon bundle.

The HOUSteons were fabricated using the aforementioned 12% PCL solution and 1393 bioactive glass fibers. The glass fiber was first wiped with isopropyl alcohol three times before being placed in the stationary holder and the ends adhered with tacky glue for each experiment, with the setup shown in Figure 1. The working distance was approximately 11 cm, with the mandrel–mandrel distance of 8 cm and a rotation speed of 169 rpm in opposite directions. With −15 kV on the needle and +15 kV on each of the mandrels, the PCL solution was extruded at 3 mL/hr. The deposited volume was approximately 0.25 mL per HOUSteon. The HOUSteon bundles were fabricated with similar parameters, except 8 HOUSteon were placed on the glass holder instead of a glass fiber. The resultant scaffolds are shown in Figure 2.

### 2.4. SEM, FFT, Fiber Diameter

SEM was performed on the scanning electron microscope model EVO LS15 (Carl Zeiss Microscopy, Cambridge, UK). The images were taken at 10–15 kV and 10–12 pA. Fast-Fourier transform (FFT) was performed using image-processing software (ImageJ, LOCI, University of Washington, Seattle, WA, USA) after performing radial sum intensities using the Oval Profile Plot plugin (author, Bill O’Connell) to obtain alignment measurements. Manual angle measurements were performed using image-processing software (ImageJ), validating the FFT angles. The diameters of 50 random fibers were manually measured throughout 2 images, by using image-processing software (ImageJ).

### 2.5. Mechanical Testing-3pt Bending

Mechanical testing was performed on an MTS Criterion model 42 (MTS Systems Corporation, Eden Prairie, MN, USA) using a 3-point bending apparatus with a support span of 1 cm. The strain rate used was 0.01 mm/s. 

### 2.6. Porosity

Porosity was determined through a version of the Archimedes method. A 10 mm biopsy punch was taken from the PCL control or a 2 cm long HOUSteon. The dry weight for each scaffold was measured, followed by a 5-second soak in 200 proof ethanol and then the wet weight was measured. The porosity (n) was found using the following equation:

(1) $$n=\frac{V_{eth}}{\left(V_{eth}+V_{PCL}\right)}\times 100$$

where *V_eth_* is the volume of ethanol intruded into the scaffold punches and *V_PCL_* is the volume of PCL fibers. The volume of ethanol intrusion was found by calculating the ratio of the observed change in mass between wet and dry weights, using the density of ethanol (0.789 g/cm^3^). The volume of PCL fibers was determined by taking the ratio of dry mass of the scaffolds and the density of PCL (1.145 g/cm^3^). To compensate for the non-porous glass fiber in the HOUSteons, the mass of the glass fiber with the same dimensions was subtracted from the dry mass. The variables *V_eth_* and *V_PCL_* were determined in the following manner, where ρ is the density:

(2) $$V_{eth}=\frac{\left(M_{wet}-M_{dry}\right)}{\rho_{eth}}\;\;\;V_{PCL}=\frac{M_{dry}}{\rho_{PCL}}$$

### 2.7. Cell Cytotoxicity

The PCL was punched out using a 10 mm diameter biopsy punch, whereas the glass and HOUSteon fibers were cut in 1 cm increments. All scaffolds were UV sterilized for 30 min, flipped, and UV sterilized for another 30 min. The scaffolds were placed in 48 well plates with cloning rings on top, and 400 μL of supplemented high glucose DMEM (1% penicillin–streptomycin and 10% fetal bovine serum) was added. Four glass and HOUSteon fibers were placed in each well. At days 1, 2, and 4, 200 μL of the media was removed, saved, and replaced with fresh media. Empty wells of media were used as controls. These timepoints of used media were added to 200 μL of fresh media containing 50,000 osteoblast-like cells from a passage 5 osteosarcoma cell line (MG63). After 3 days of culturing, a cell proliferation MTS assay (Celltiter 96 Aqueous Nonradioactive Cell Proliferation Assay, Promega, Madison, WI, USA) was performed to establish cell number. In short, the media were replaced with 200 μL of fresh media with 40 μL of 20:1 MTS:phenazine methosulfate (PMS) solution added. After an hour at 37 °C, 100 μL was removed from each well and placed in a 96 well plate, with the absorbance measured at 490 nm.

### 2.8. Cell Adhesion

The PCL was punched out using a 10 mm diameter hole punch, whereas the glass and HOUSteon fibers were cut in 1 cm increments. The wells containing no scaffold were used as controls. The scaffolds were UV sterilized as before and placed into 48 well plates with cloning rings on top. Four glass and HOUSteon fibers were placed in each well. 200 μL of media containing 100,000 MG63 cells were added to each well, and allowed to culture for 3 h. Scaffolds were removed and placed in a new 48 well plate, where the aforementioned MTS/PMS assay was performed. When the HOUSteons and glass fibers were cut prior to the assay, pieces in between the cut were saved and imaged with the SEM. The measured diameter and length of those scaffolds were used to calculate and normalize to the surface areas, since the PCL was much larger (~25 mm^2^ for HOUSteon compared to the top surface area for the PCL of ~78 mm^2^). 

### 2.9. Confocal Imaging

All confocal images were taken using Olympus FV1000 laser scanning confocal microscope. All cells were stained with 4′,6-diamidino-2-phenylindole (DAPI). Some cells were stained with Celltrace Far Red cell proliferation, which covalently binds to cellular amines and can indicate the extending protrusions of cells.

### 2.10. ICP-OES Glass Conversion

The fibers were cut into 1 cm long segments, approximately 1 mg of glass per sample. The samples were submerged in 1 mL PBS in 37 °C incubator for the 1–4 weeks, creating a 1 g/L ratio. The solutions were removed each week and stored in the −80 °C freezer until use. The solutions were brought to 10 mL with an end concentration of 2% HNO_3_ in deionized water prior to analysis. The samples were analyzed with an Optima 8300 inductively coupled plasma–optical emission spectrometer (ICP-OES, Perkin-Elmer, Waltham, MA, USA). 

### 2.11. Statistical Analysis

SPSS software (IBM, Armonk, NY, USA) was used to determine statistical significance with an alpha value of 0.05. Independent sample t-tests were used to compare between two variables. One-way ANOVA with a Tukey post-hoc analysis was performed to evaluate significance between multiple samples.

## 3. Results

### 3.1. Design and Optimization of the Modified Air-Gap Electrospinning Setup

The design of the air-gap electrospinning apparatus is composed of both stationary and rotating parts, as shown in Figure 1a. This design keeps the glass fiber stationary, allowing the electrospun fibers to wrap around it, which is demonstrated in Figure 1b. When the glass fiber is not held in place, then typically the slower side of the mandrel entraps the glass fiber forcing it to rotate with that side. This ultimately ends up with half of the polymer fibers not wrapping around the glass fiber. Using this motor-gear setup allows for the mandrel to rotate around the stationary glass fiber, without the motors being shocked from the voltage applied to the mandrels. 

Furthermore, the flat-cone shaped mandrel was chosen over the flat plate stereotypical used for air gap electrospinning in order to decrease the effective diameter as much as possible. Decreasing the mandrel size causes a lower angle of fiber wrapping, making the polymer fibers more aligned to the glass fiber. The cone attracted the fibers more efficiently than its flat-plate equivalent, while only adhering to the tip of the cone. This allows for a significantly smaller effective diameter of the mandrel, such as the 4 mm cone-tip that was used in this design.

One drawback with air gap electrospinning is that the fibers will deposit only on empty mandrel sites, preventing fibers from depositing when the mandrel is covered. Additional fibers will deposit on only one side of the mandrel, not deposit on either side, or randomly deposit onto the aligned fibers. When a fiber is deposited on this rotating mandrel, it will wrap around the glass fiber starting at the center and work its way towards the mandrels. With the current design involving the protruded stationary glass holder from the mandrel, the fibers will eventually wrap around the holder until enough tension pulls the fiber off the mandrel. This frees up space on the mandrel, allowing more fibers to deposit. This process can be repeated continuously until some other failure occurs, such as the glass fiber breaking. This allows for the number of layers of wrapped PCL to be controllable while not being limited by the mandrel not having available spots to deposit on. Initial designs had the stationary glass holder inside of the cone, but the polymer fibers pull the glass fiber out of its holder, halting electrospinning.

**Figure 3:**
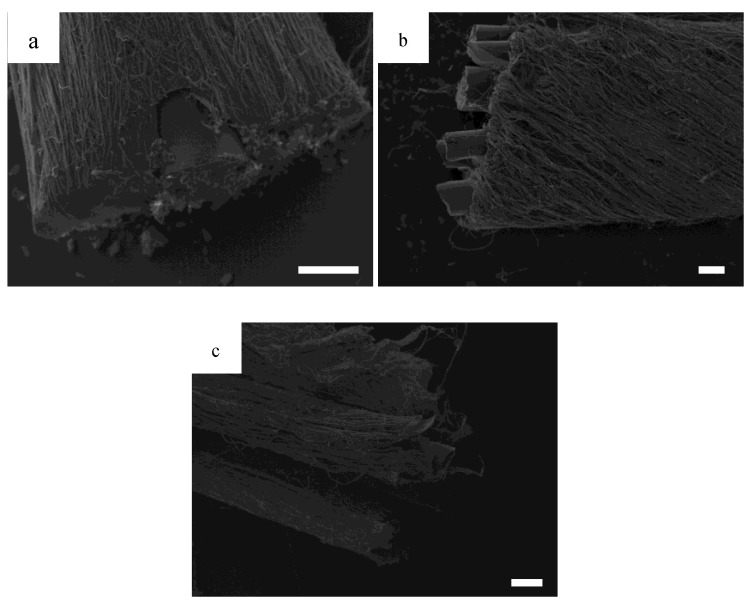
SEMs of (**a**) a ~150 μm diameter glass fiber wrapped in electrospun nanofibers to a total of ~500 μm diameter; (**b**) a bundle of eight ~200 μm diameter homologous and uniform synthetic osteons (HOUSteons) wrapped together to a total diameter ~800 μm, with (**c**) the outer layer peeled back. Scale bar is 100 μm.

To demonstrate the capability for this design to continuously electrospin, Figure 3a shows a HOUSteon that was allowed to electrospin for longer, resulting in a diameter around 500 μm with a glass diameter around 150 μm. In Figure 3b,c, eight typical HOUSteons with a diameter of ~200 μm were adhered together and then placed in the same modified air-gap electrospinning setup, undergoing the same electrospinning process to form a bundle of HOUSteons. In Figure 3b, the outermost casing of electrospun fibers are shown, with the glass fibers exposed. In Figure 3c, the outer casing was peeled back, revealing the maintained HOUSteon structure when incorporated into a bundle.

### 3.2. SEM, FFT, Fiber Diameter

**Figure 4:**
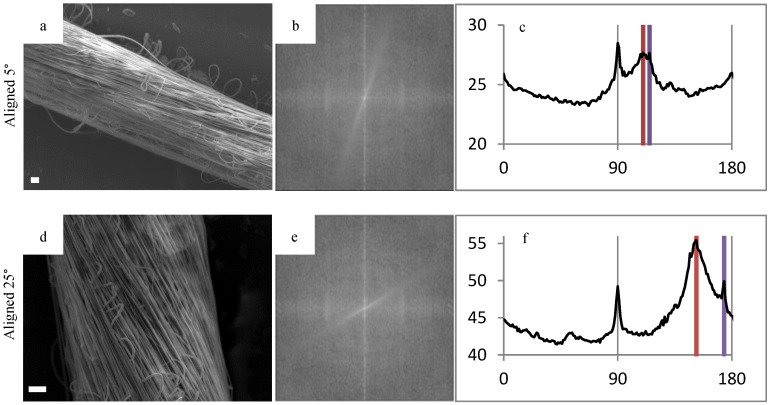

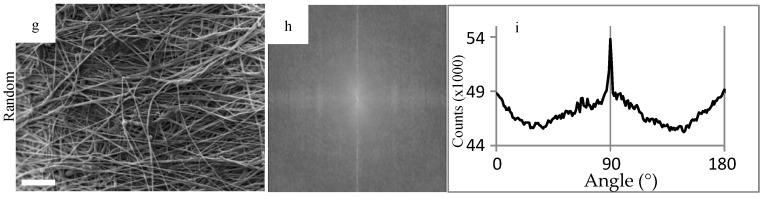
Fast Fourier transform (FFT) of representative scaffolds to demonstrate the tailorability in fiber alignment. FFT was used on (**a,d,g**) SEM images in order to gain the respective (**b,e,h**) FFT output images and (**c,f,i**) pixel intensity plots against the angle of acquisition. The alignment angle was determined through subtracting the peaks, resulting in (**a,b,c**) 5°, (**d,e,f**) 25°, and (**g,h,i**) random. Scale bar is 20 μm.

To demonstrate the range of angles that the polymer fibers can wrap around the glass fiber, FFT was performed on representative images of different diameter mandrels in Figure 4. The pixel intensity plots showed two distinct peaks, which correlates to be the orientation of the individual polymer fibers as well as the orientation of the HOUSteon as a whole. The difference between these peaks yields the angle of polymer fiber wrapping around the glass fiber. These values were validated by using image-processing software (ImageJ) to measure the respective angles and comparing them to the FFT results. 

**Figure 5:**
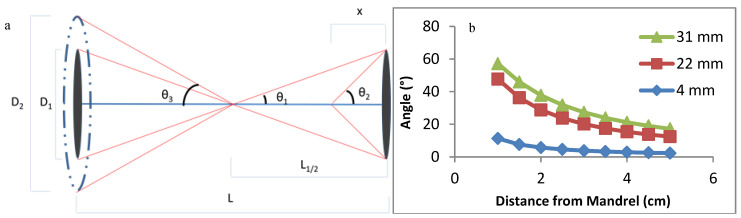
(**a**) Diagram and Equation (3) of a theoretical polymer fiber wrapping around the glass microfiber, where D is the diameter of the mandrel, θ is the angle the fiber wraps around the glass fiber, L is the length between mandrels, and x is the distance from the mandrel. (**b**) Theoretical fiber angles with mandrel diameters of 4, 22, and 31 mm at varying distances.

The alignment angle of the polymer primarily depends on the diameter of the air-gap mandrels and the distance from the mandrel, as summarized in Figure 5 and Equation (3) Figure 5b demonstrates that reducing the diameter of the mandrel is the most effective way of reducing the angle, as well as reducing the angle variance along the length of the HOUSteon. This not only increases consistency between samples, but also along the distance of each HOUSteon. Hence, the 4 mm cone-shaped mandrel was chosen for further testing.

(3) $$\theta ={\mathrm{Tan}}^{-1}\left(\frac{D}{2x}\right)\;\mathrm{or}\;x=\frac{D}{2\mathrm{Tan}\left(\theta \right)}$$

### 3.3. Mechanical Testing-3pt Bending and Porosity

**Figure 6:**
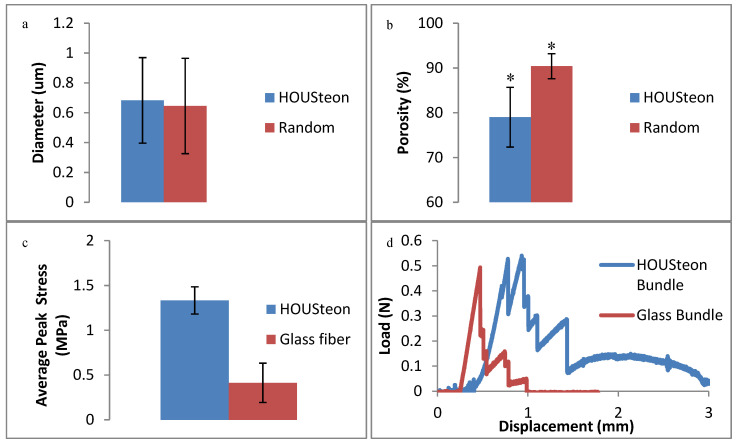
Physical characterization of (**a**) average PCL fiber diameter and (**b**) porosity between the HOUSTeon and randomly aligned PCL. The 3-point bending of (**c**) the peak stress between the HOUSteon and bare glass at 0.01 mm/s. (**d**) A representative 3-point bending load-displacement curve comparing a bundle of eight HOUSteons and 8 bare glass. * denotes significant difference (*p* < 0.05).

When comparing the PCL fibers in Figure 6a,b, the porosity decreases to 79% when the fibers were aligned while still maintaining the same fiber diameter. Aligning and wrapping the fibers closes the pores by forcing the fibers to be adjacent to one another, whereas the randomly oriented fibers are allowed to deposit freely. In Figure 6c, the addition of the PCL fibers had no significant effect on the peak stress applied to the glass fibers. Although they achieved similar peak stresses, the PCL in the HOUSteons maintained the load due to its tensile, viscoelastic deformation after glass fracture. This is further demonstrated in Figure 6d, where bundles of eight HOUSteons were compared to eight glass fibers bound at the ends. This is most evident with the sustained load after all of the glass fibers fractured, but can be seen throughout by the increased recovery after at least one of the glass fibers breaks, indicated by vertical drops. 

### 3.4. Cell Cytotoxicity and Adhesion

**Figure 7:**
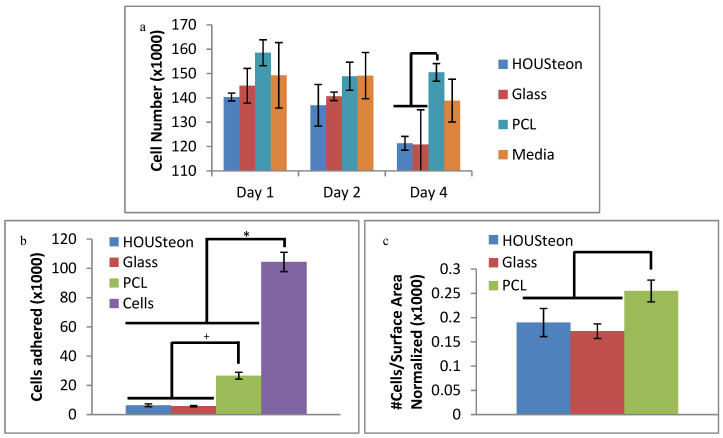
Cell assays of (**a**) in vitro cytotoxicity and (**b,c**) adherence. (**c**) Cells adhered to the scaffolds were normalized to the available surface area on the scaffolds and to the cell control. * and + denote statistical significance (*p* < 0.05).

In Figure 7a, the released particles from all of the different scaffolds had no significant cytotoxic impact on the cells, as compared to the fresh media control. The decrease in cells of the glass-containing scaffolds compared to PCL can be attributed to pH alkalization during glass conversion under these static conditions [39]. This is mitigated through dynamic cell culturing conditions, which are more representative of in vivo conditions, or through preconditioning the scaffolds. The cells adhered from Figure 7b were normalized to the surface area available to the cells and to the cell control, resulting in Figure 7c. This was to compare the number of cells adhered to the estimated amount of available material, since the PCL punches had around three times more surface area exposed than the HOUSteons. After normalization, the amount of cells adhered is comparable to the randomly oriented PCL, indicating the cells adhere with similar affinity to the PCL as well as the PCL on the HOUSteons. It is worthwhile to note that more accurate results can be obtained by increasing the amount of HOUSteons used to make it equivocal to the PCL, but the relative amount of cells adhered demonstrates its potential.

### 3.5. Confocal Imaging

**Figure 8:**
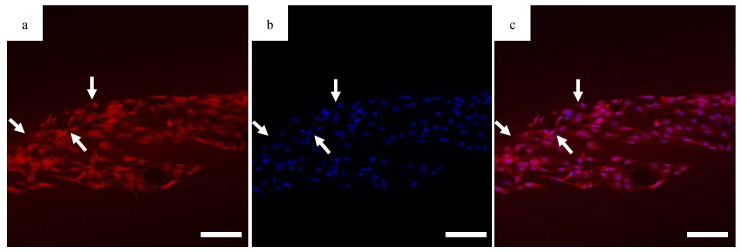
Confocal images of the (**a**) cytoplasm, (**b**) DAPI, and (**c**) composite image for MG63 cells on the HOUSteon. The HOUSteon fractured prior to cell adhesion, and cell migration is visible on the bridging PCL fibers (arrows). Scale Bar is 100 μm.

**Figure 9:**
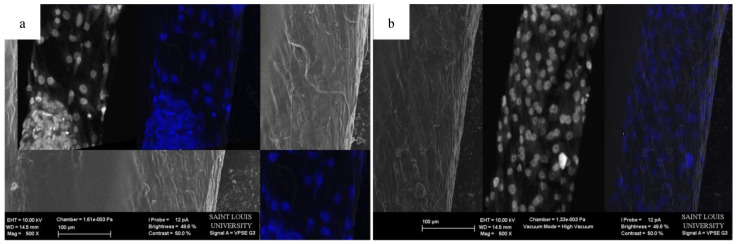
Representative SEM images of a HOUSteon with overlaid confocal images stained with DAPI of (**a**) an aberrant fiber and (**b**) aligned fibers.

The migration of the cells is sensitive to fiber alignment and responds to aberrant fibers. The cells are capable of compensating for defects in the scaffold, as demonstrated in Figure 8 when the cells used the bridging PCL to cross between the severed halves of the HOUSteons. This is beneficial for glass fractures, but can be inconvenient when aberrant fibers are deposited on the scaffold during manufacturing, such as in Figure 9a. The horizontal fiber hindered cell migration, forcing cells to either go around or cross over it. While not ideal, the cells were still capable of continued migration along the HOUSteon. Furthermore, scaffolds without any large defects in Figure 9b allowed cell migration to achieve a near confluent layer, signifying the ideal interaction between the cells and the PCL on the HOUSteon. 

### 3.6. ICP-OES Glass Conversion

**Figure 10:**
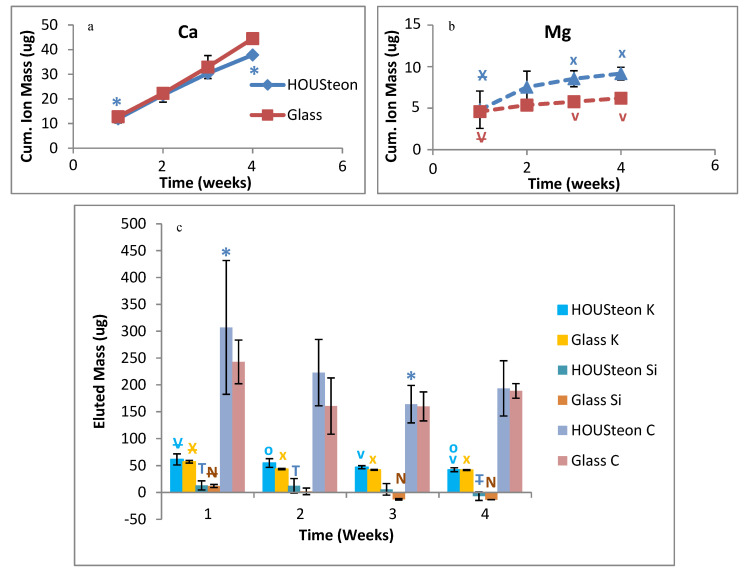
ICP-OES of the cumulative ion mass eluted of (**a**) calcium and (**b**) magnesium. (**c**) The eluted mass of potassium, silicon, and carbon. *, V, X, T, N, and O denote statistical significance, with strikethrough indicating parent significance to respective letters (*p* < 0.05).

Ion release measurements from ICP-OES demonstrate that the polymer wrapping does not inhibit the glass conversion. Similar calcium elution was observed between scaffolds in Figure 10a, but calcium is incorporated into HA. Magnesium is an ion that is in neither PBS nor in normal HA, with both scaffolds having increased magnesium elution at similar rates, as shown in Figure 10b. Therefore, the eluted magnesium is indicative of glass degradation. Both scaffolds showed similar trends of eluted mass for potassium, silicon, and carbon, as shown in Figure 10c. While the elution of silicon is indicative of glass degradation, the conversion to HA utilizes the porous silica layers to maintain the original structure of the phosphate-based glass. 

## 4. Discussion

The creation of a synthetic scaffold that is osteoconductive and osteoinductive, while promoting angiogenesis, is necessary to replace the current gold standard of autografts [40]. Most of the current designs for biomimetic scaffolds focus on the material aspect of bone, by using collagen derivatives and calcium phophate-based ceramics as a replacement for bone’s natural elasticity and mechanical strength [41]. Animal-extracted collagen and gelatin have high tissue regeneration potentials due to their resorbability, low antigenicity, and high cytocompatibility [42,43]. Bioactive ceramics, HA, calcium phosphates, and glasses are typically used to provide better mechanical strength and bone formation than collagen derived materials alone [44]. Incorporating well-characterized growth factors or cultured cells into these scaffolds have been shown to increase overall bone formation as well [44]. 

These composite scaffolds have a variety of structures, with some common forms being three-dimensional structures [42,45], hydrogels [46], and dry powder [47]. The formation of these scaffolds are typically through solvent [48], freeze-drying [49], gas-foaming, or electrospinning [50] followed by a crosslinking method for solidification of the polymer. Furthermore, the incorporation of the ceramics can be classified as either within the scaffold during fabrication or deposited onto the surface. Combining scaffold methods, adjusting the materials used, and incorporating additional materials have been shown to adjust the properties of the scaffolds [48]. Unfortunately, current scaffolds are not as efficient at bone healing as the autograft gold standard, one common problem with biomaterials being the slow implant integration [49]. Copying the structure of naturally occurring tissues is one strategy to improve integration with adjacent tissues [49].

The design in this study was focused on mimicking osteons with concentric polymer layers surrounding haversian canals while creating radial porosity for nutrient diffusion. The number of electrospun layers, which is analogous to the bone matrix lamellae, is contingent on the volume electrospun. This causes the HOUSteons to have dimensions comparable to native osteons [8]. The alignment of the electrospun fibers allows for increased cell migration along the length of the HOUSteon, whereas the degradation of the glass microfiber is designed to promote blood vessel ingrowths. Maintaining the high porosity from normal electrospinning is essential for not hindering the glass conversion using this setup, since the water must infiltrate through the electrospun layer, hydrolyze the glass, and begin its conversion process. The alignment of the polymer fibers is worth this slight decrease in porosity, since 80% porosity is sufficient for water infiltration and glass conversion, which is demonstrated by ICP. All of the eluted ions showed similar elution between the bare glass and the HOUSteon. This reduced porosity is expected to decrease the cellular infiltration through the thickness of the HOUSteon, but the aligned fibers increase directional cell migration along the length of the HOUSteon. Alternatives to increase porosity include increasing the fiber diameter or introducing sacrificial fibers.

A common criticism of using ceramics or glass as an implant is that the breakage results in catastrophic failure, rendering the implant functionally useless afterwards. Although not comparable to native cortical bone of 100–150 MPa compressive strength or 20–40 MPa tensile strength [51], wrapping the glass with the polymer fibers holds the glass in place, maintaining some structural integrity after the glass breaks. This overcomes its catastrophic failure by instead having viscoelastic breakage, with no significant difference in peak stresses. More importantly, the cells were able to cross the polymer fibers after breakage, bridging the gap and progressing forward. This maintains the bioactivity of the scaffold even if it breaks.

The initial degraded components of the HOUSteon were not significantly more cytotoxic to the cells than plain media. Although in vitro analysis of bioactive glass can be contradictory and cytotoxic at times due to static culturing conditions and different cytotoxicity methods, bioactive glass has positive results in vivo [24,52]. The degradation of the glass creates a nearby acidic environment, but the in vivo environment should dilute and remove the acidic byproducts [24,53]. Additionally, the normalized cell adherence of the HOUSteons was comparable to PCL. This is expected since the composition and diameters of the PCL fibers are the same, with the main superficial difference between them being the available surface area.

One main component of this design is to increase cell migration due to the aligned fibers. Previous work has already demonstrated that aligned electrospun fibers enhance directional cell migration [13,14,15,16,17,18,19]. Initial cell seeding showed the cells extending parallel to the fibers, signifying unidirectional orientation and thus increased cell migration. Due to the weakness of a single HOUSteon as well as the expected mechanical strength decreasing during degradation, breakage of some HOUSteons is expected. Figure 8 demonstrates that a broken HOUSteon is capable of cell migration along the stretched fibers between the broken pieces. This allows for continued cell activity and bone formation despite HOUSteon breakage. Additionally, the cells were affected by aberrant fibers, slowing the directional migration of cells when encountered. Because of this sensitivity though, the cells also elongate and respond to the normal, parallel fibers. These curved, aberrant fibers, which deviate from aligned fibers, were found to occur during electrospinning when the fiber is longer than the average fiber. This causes adjacent fibers to entrap it into the HOUSteon during wrapping. Guaranteeing there is an available spot on the mandrel by reducing the flow rate, or accelerating the fibers quicker to the mandrels by adjusting their charge, is expected to mitigate aberrant fibers.

Since the purpose of the HOUSteon is to facilitate the migration and proliferation of the osteogenic cells, it is imperative that blood vessels infiltrate into the scaffold to provide the necessary nutrients. The HOUSteon design can easily change its composition to match well-researched materials and constituents relevant for bone and blood vessel growth. For example, incorporating already established pro-angiogenic ions into the glass, such as copper and cobalt, will induce blood vessel growth [54]. Adding calcium or pro-angiogenic growth factors into the electrospun fibers allows for a release rate separate from the bioactive glass. Furthermore, substituting borate for silicate [26] or increasing the ionic radius of network modifiers, such as replacing K_2_O for Na_2_O [27], will speed up the degradation rate to more closely match the ingrowths of blood vessels. Similarly, increasing the hydrophilicity of the electrospun fibers should help increase the influx of water towards the glass. Therefore, HOUSteons provide the structure similar to osteons, with the polymer and bioactive glass compositions capable of being easily altered to further promote regeneration.

## 5. Conclusions

In this study, a synthetic scaffold was designed to mimic the structure and dimensions of an osteon, the individual unit of cortical bone. These aligned electrospun fibers held the bioactive glass fiber together, while not inhibiting glass conversion. Inversely, the degraded ions from the glass did not prevent cell adhesion or cause significant cell death. The versatility of this design allows for the glass and polymer fibers to be replaced with more osteogenic and angiogenic materials, with further incorporation of nanoparticles or growth factors to facilitate growth. Utilizing this biomimetic scaffold design with more bioactive materials, this synthetic scaffold aims to replace autografts to heal critical size bone defects.
